# Livestock and avermectins in sub-Saharan Africa: a restricted systematic review of the impacts on productivity and documentation of resistance

**DOI:** 10.1017/S0031182025100280

**Published:** 2025-05

**Authors:** Cassidy Rist, Rose Zheng, Lauren Maghak

**Affiliations:** Department of Population Health Sciences, Virginia-Maryland College of Veterinary Medicine, Virginia Tech, Blacksburg, VA, USA

**Keywords:** anthelmintic resistance, avermectins, endectocide, ivermectin, livestock, malaria

## Abstract

There is growing interest in using avermectins in livestock as a vector control tool for mosquitoes involved in the transmission of human malaria in sub-Saharan Africa (SSA). If implemented, the potential health and productivity impacts across the livestock sector would need to be considered, as avermectins are already commonly used in veterinary medicine to treat gastrointestinal helminths and parasitic insects. Here we present the results of a restricted systematic review that summarizes what is known about the effects of avermectins on cattle and swine productivity in SSA and the presence of avermectin resistance in endo- and ectoparasites of importance in these species. A total of 583 unique journal articles were identified using key search terms in 3 databases: Agriculture, Life, and Natural Sciences Databases from ProQuest, CAB Abstracts and Scopus. Ten articles met the criteria for inclusion on impacts on productivity and 4 met the inclusion criteria related to avermectin resistance. All studies documenting impacts of avermectins on productivity were performed using ivermectin in cattle. Generally, these showed a positive significant effect on growth rates. Resistance to avermectins was documented in 2 of the 4 included articles. Considering the extensive literature documenting resistance to avermectins in other areas of the world, our findings may reflect a paucity of studies on the subject in SSA. The authors conclude that additional research is needed to quantify the potential benefits and challenges to the livestock sector of using avermectins for malaria control across different production systems, and in a variety of ecological settings.

## Graphical abstract

**Figure:**
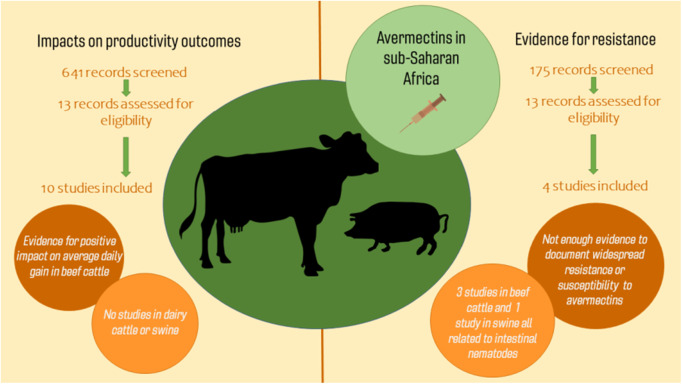

## Introduction

Avermectins are commonly used around the world in cattle, small ruminants and swine to treat gastrointestinal nematodes and many ectoparasites. Ivermectin is perhaps the most widely used and well-known of the available avermectins, but other examples labelled for use in livestock include eprinomectin and doramectin. Although there is strong evidence that treating livestock with avermectins to control parasites improves animal productivity, most of the research has been performed in Europe and the USA (Nødtvedt et al. 2002; Cringoli et al. 2009; Rehbein et al. 2003, 2016; Kunkle et al. 2013; Verschave et al. 2014). Location of the research is important as animal genetics, environmental conditions and production systems (e.g. intensive vs. extensive) likely influence the relationship among parasite prevalence, impacts on productivity and follow-on economic consequences of production losses (Lamy et al. 2012).

Currently, there is significant scientific interest in using ivermectin in mass drug administration (MDA) campaigns in humans and livestock as a vector control tool for mosquitoes involved in the transmission of malaria (Poché et al. 2015; Chaccour et al. 2023). This interest stems from evidence that *Anopheles* mosquitos that feed on ivermectin-treated blood sources die or exhibit reduced reproductive success (Poché et al. 2015; Pooda et al. 2015; Lyimo et al. 2017), thereby serving to reduce the mosquito population. In areas where malaria vectors exhibit partial zoophagy (blood feeding on animals), the use of ivermectin in livestock in addition to humans serves to cover a greater proportion of blood sources available. There are several field studies underway to determine if this approach will have the anticipated effects of reducing mosquito populations and lowering malaria transmission.

Even if successful relative to malaria control, there are other benefits and risks to consider that arise with the delivery of ivermectin in livestock populations (Ruiz-Castillo et al. 2022). For example, treated animals also derive health benefits from a reduced parasite burden, which can translate into increased productivity and follow-on economic and nutritional benefits for livestock owners and the community (Rist et al. 2015; Strydom et al. 2023). However, resistance to ivermectin and other avermectins in livestock species is a growing concern and has been well documented for decades across various parasites of importance to livestock health (Shoop, 1993; Kaplan, 2004; Sutherland et al. 2011; Wolstenholme et al. 2012; Kotze et al. 2016; Rodriguez-Vivas et al. 2017). If successful as a novel vector control tool, the increase in ivermectin use in livestock for malaria programs could contribute to the development of avermectin-resistant parasites among livestock owned by some of the most vulnerable populations. This in turn could have negative impacts on animal productivity, household nutrition and economic security.

The intent of this restricted systematic review is to summarize existing evidence on the effect of avermectins on cattle and swine productivity, and the distribution of avermectin resistance in internal and external parasites of cattle and swine in SSA. The scope was limited to cattle and swine as these are the 2 species for which studies have documented that treatment with avermectins has a negative effect on the life span and reproductive success of blood-fed mosquitoes (Ruiz-Castillo et al. 2022). In addition, the scope was limited to SSA as this is where over 90% of malaria cases occur (Venkatesan, 2024) and is the geographical area most likely to implement the use of ivermectin MDA if the strategy proves effective.

Summarizing the available evidence for impacts on livestock productivity and parasite resistance is critical to the overall evaluation of the use of avermectins in livestock for vector control – what evidence do we have and what yet needs to be determined in order to implement such strategies in a manner that promotes the benefits to livestock health, while mitigating the risks? While avermectin resistance in parasites of importance to livestock health has been extensively studied, to the author’s knowledge, no previous review has specifically focused on cattle and swine in SSA, and the small-holder livestock systems that predominate in this region of the world.

## Materials and methods

### Study protocol

This paper follows the guidelines for a restricted systematic review (i.e. rapid review) as outlined by Plüddemann et al. (2018). The original search protocol was previously published (Rist et al. 2020), so only a brief overview of the search process and inclusion criteria is described here. The only change to the published search protocol is that the search dates were updated to extend through 30 April 2024.

A pair of focal research questions were addressed in this review and are outlined below:

- Research Question 1: *What are the effects of avermectins on cattle and swine productivity in sub-Saharan Africa (*SSA*), where productivity includes measures such as growth rate, reproductive success or milk production?*
- Research Question 2: *What is known about the distribution of avermectin resistance in parasites of cattle and swine in SSA?*

The databases used were CAB Abstracts from Cab Direct, Scopus and the Agriculture, Life, and Natural Sciences Databases from ProQuest (a federated search comprised of databases within Virginia Tech’s subscriptions).

To address question one, the review protocol was developed based on the PICO framework, with inclusion criteria defined as follows:

- population: a population of cattle and/or swine in SSA;
- intervention: treatment of livestock for endo- or ectoparasites utilizing ivermectin, eprinomectin or doramectin;
- comparison: compared against a control group, or group treated with a rival anti-parasitic drug and
- outcome: a change in productivity measured as alterations in growth rate, reproductive success or milk production (cattle only). Outcomes were later expanded to include a reduction in cutaneous lesions and associated tissue trimming caused by parasites traditionally susceptible to avermectins.

To address question 2, the review protocol was based on the PEO framework, with inclusion criteria defined as follows:

- population: a population of cattle and/or swine in SSA;
- exposure: treatment with ivermectin, eprinomectin or doramectin and
- outcome: measure of avermectin resistance in an endo- or ectoparasite.

## Results

### Study selection

#### Research question 1 – Livestock productivity

**Figure 1.**
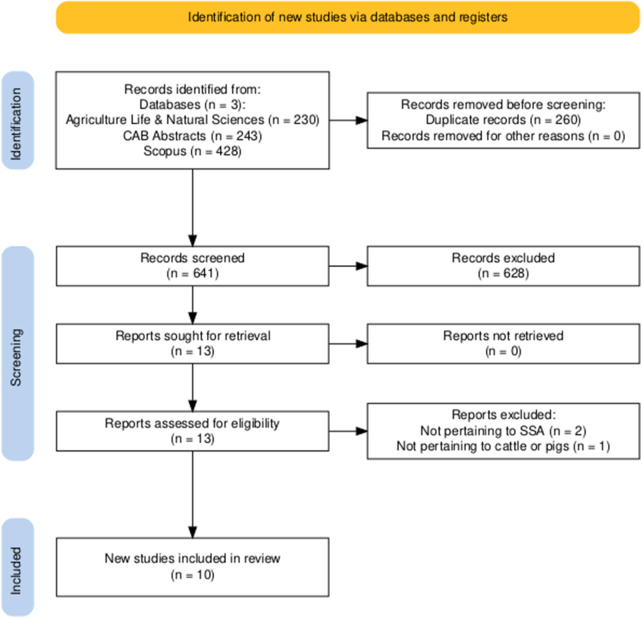
Objective 1 flow chart based on PRISMA guidelines, illustrating the total number of records (research articles) identified on initial search, and the number of records filtered out with each stage of the selection process. The figure was created using Haddaway et al. (2022).

A total of 901 articles were identified during the search process (Figure 1). Of this number, 260 were duplicates, leaving 641 articles available for initial screening. After initial screening, 13 articles were retrieved and reviewed in full. Three articles were excluded due to not pertaining to SSA (*n* = 2) or not being pertinent to cattle or pigs (*n* = 1), leaving a total of 10 articles included in the review. The publication dates of included articles spanned from 1983 to 2001.

#### Research question 2 – ivermectin resistance

**Figure 2.**
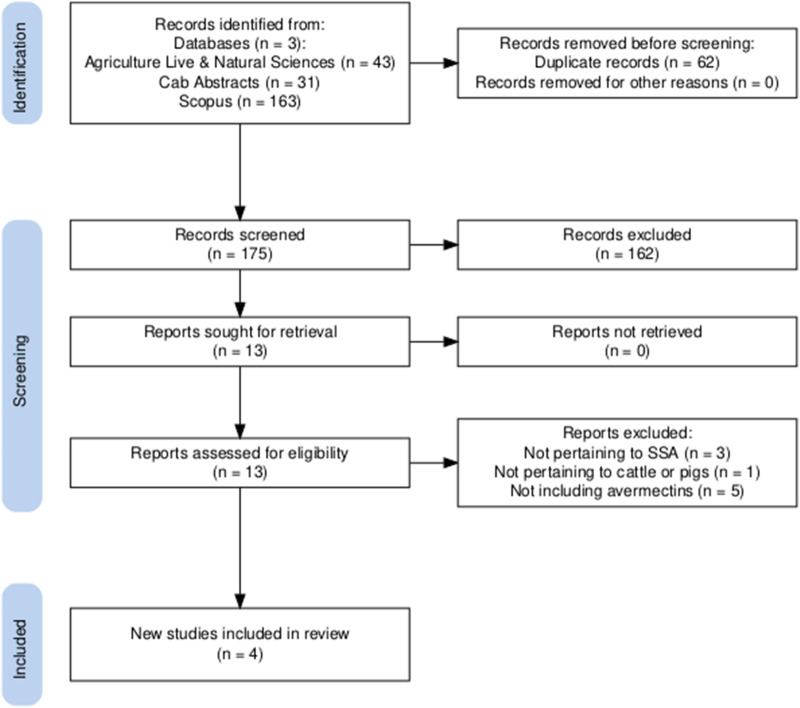
Objective 2 flow chart based on PRISMA guidelines, illustrating the total number of records (research articles) identified on initial search, and the number of records filtered out with each stage of the selection process. The figure was created using Haddaway et al. (2022).

A total of 237 articles were identified through the search process (Figure 2). Sixty-two were duplicates, leaving 175 available for initial screening. After initial screening, 13 articles were retrieved and reviewed in full. Nine articles were excluded due to not pertaining to SSA (*n* = 3), not including avermectins (*n* = 5) and not being pertinent to cattle or pigs (*n* = 1), leaving a total of 4 articles included in the review. The publication dates of included articles spanned from 2012 to 2017.

### Cattle and swine productivity

The 10 included studies were carried out in the following SSA countries: Kenya (*n* = 2), South Africa (*n* = 3), Sudan (*n* = 1), Zambia (*n* = 1) and Zimbabwe (*n* = 3) (Table 1). All studies were conducted on beef cattle, and there were no articles including dairy cows or swine that met the eligibility criteria. Additionally, there were no studies included that pertained to ectoparasites. Animals in all studies were naturally infected before the studies began, and the majority of gastrointestinal helminths identified were common species known to infect cattle globally (*Haemonchus, Trichostrongylus, Cooperia, Oesophagostomum* and *Stronglyloides*).

**Table 1.** Summary of the studies (*N* = 10) describing the effect of avermectins on cattle and swine productivity in sub-Saharan Africa

Authors	Year	Title of study	Country	Type of avermectin, dose and delivery	Parasite species identified	Comparison group	Results
Swan et al.	1983	Efficacy of ivermectin against *Parafilaria bovicola*	South Africa	Ivermectin 200 mcg kg^−1^, PO or SC	*Parafilaria bovicola*	Untreated	88.2% reduction in the number of subcutaneous lesions, 98.7% reduction in the total lesion area and 98.8% reduction in weight of tissue trimmed from carcasses 83 days post-treatment with SC delivery. No effect in cattle treated orally.
Soll and Carmichael	1984	The influence of pre-slaughter treatment with ivermectin on *Parafilaria bovicola* infestation in cattle in Zimbabwe	Zimbabwe	Ivermectin 200 mcg kg^−1^, SC given 50 or 70 days pre-slaughter	*Parafilaria bovicola*	Untreated	*50 days*: 57.6% reduction in lesion size and 71.7% reduction in trimmed tissue. *70 days*: 93.3% reduction in lesion size and 92.4% reduction in trimmed tissue.
Abdalla	1989	Effects of endoparasites on the growth rate of Sudanese sheep and cattle	Sudan	Ivermectin 200 mcg kg^−1^, SC	*Haemonchus, Trichostrongylus, Cooperia, Oesophagostomum, Stronglyloides* and *Chabertia*	Untreated	No statistical difference in liveweight gain between treated and control cattle over a 28-day period.
Swan et al.	1991	Efficacy of ivermectin against *Parafilaria bovicola* and lesion resolution in cattle	South Africa	Ivermectin 200 mcg kg^−1^, SC given 15, 30, 50 or 70 days pre-slaughter	*Parafilaria bovicola*	Untreated	Cattle treated 70 days pre-slaughter had significant reductions in the number and surface area of lesions (1 lesion), and the weight of trimmed tissue (no tissue trimmed) as compared to controls. There was no significant difference in cattle treated at <70 days as compared to control.
Soll et al.	1991	Ivermectin treatment of feedlot cattle for *Parafilaria bovicola*	South Africa	Ivermectin 200 mcg kg^−1^, SC on day 0, 21 or 54 upon entry into a feedlot	*Parafilaria bovicola*	Untreated	83.3% reduction in lesion area and 89.9% reduction in mean mass trimmed from carcass across all groups as compared to control when slaughtered >84 days post-entry.
Duncan and Forbes	1992	Comparison of productivity and economic benefits of strategic anthelmintic use in young beef cattle in Zimbabwe	Zimbabwe	Ivermectin 200 mcg kg^−1^, SC on days 0, 194, 354 and 391 (4 doses)	No faecal analysis performed	(1) Untreated (2) Levamisole hydrochloride 7.5 mg kg^−1^, PO on Days 0, 194, 354 and 391 (4 doses) (3) Oxfendazole 2.5 mg kg^−1^, PO on Days 0, 194, 354 and 391 (4 doses)	Increase in the mean total weight gain over 480 days across all treatment groups as compared to untreated control. Ivermectin-treated group gained 40 lbs more than the control group and 20 pounds more than the oxfendazole group.
Vassilev	1993	Activity of ivermectin and albendazole in the control of gastrointestinal nematode parasites and growth performance of two-year-old beef cattle	Zimbabwe	Ivermectin 200 mcg kg^−1^, SC every 10 weeks for 3 doses; ivermectin 200 mcg kg^−1^, SC 2 doses given 20 weeks apart	*Cooperia, Haemonchus placei* and *Oesophagostomum*	(1) Untreated (2) Albendazole 7.5 mg kg^−1^ PO every 10 weeks for 3 doses	Over 1 year, 3 doses of ivermectin showed 23.4 kg greater average live mass gain as compared to control, 25.7 kg greater gain as compared to 2 doses of ivermectin and 10.5 kg greater gain as compared to the albendazole group; cattle treated with 3 doses of ivermectin had an average daily gain of 0.212 kg day^−1^ in treated vs. 0.148 kg day^−1^ in untreated cattle. *2 doses ivermectin*: no significant difference as compared to untreated.
Meeus et al.	1997	Comparison of the persistent activity of ivermectin, abamectin, doramectin and moxidectin in cattle in Zambia	Zambia	Abamectin 200 mcg kg^−1^, SC; doramectin 200 mcg kg^−1^, SC; ivermectin 200 mcg kg^−1^, SC	*Cooperia* and *Haemonchus*	Albendazole 7.5 mg kg^−1^, PO	No statistical difference in the total body weight gain was found between any of the groups over the 84-day study period, despite a significant reduction in faecal egg count in the avermectin groups compared to the albendazole group.
Munyua and Ngotho	1998	Efficacy of ivermectin delivered from a sustained-release bolus against gastrointestinal nematodes in field grazing calves in Nyandarua district of Kenya	Kenya	Ivermectin as a sustained-released bolus designed to deliver 12 mg day^−1^ for 135 days	*Haemonchus placei, Trichostrongylus axei, Cooperia* spp., *Nematodirus helvetianus, Oesophagostomum radiatum* and *Trichuris* spp.	Untreated	Significant increase in average daily gain up to 120 days post-treatment (0.335 kg day^−1^ vs. 0.005 kg day^−1^ in controls).
Waruiru and Ngotho	2001	Influence of ivermectin and clorsulon strategic treatments on liveweight gain and helminth infections of grazing calves in Kenya	Kenya	Ivermectin 200 mcg kg^−1^, SC every 4 months; ivermectin 200 mcg kg^−1^ with clorsulon, SC every 4 months × 1 year	Strongyles (general) and *Fasciola hepatica* (not treatable by ivermectin)	Untreated	Calves treated with ivermectin alone had significantly higher average daily gains as compared to untreated controls (0.410 kg day^−1^ vs. 0.312 kg day^−1^), but the greatest gain was seen in claves treated with ivermectin and clorsulon (0.515 kg day^−1^).

Ivermectin was used in all 10 studies. One study also included abamectin and doramectin (Meeus et al. 1997), and another tested ivermectin against ivermectin with clorsulon (Waruiru and Ngotho, 2001). In 9 studies, ivermectin was delivered subcutaneously (SC) at its labelled dose of 200 mcg kg^−1^ with one study testing a novel sustained release bolus delivering 12 mg day^−1^ for 135 days (Munyua and Ngotho, 1998). Abamectin and doramectin, when used, were also dosed at 200 mcg kg^−1^. Additionally, one study compared subcutaneous delivery with an oral 200 mcg kg^−1^ dose of ivermectin (Swan et al. 1983).

Six studies evaluated the effect of ivermectin on cattle growth rates, commonly measured as total live weight gain and/or average daily gain. Of the 6 studies, 4 monitored changes in cattle weight for 4 months or longer, while 2 studies followed the treated cattle for periods less than 3 months post-treatment. The 4 studies that monitored changes in weight for 4 months or longer all delivered more than one dose of ivermectin and demonstrated a significant positive effect on cattle growth, as compared to untreated animals (Duncan and Forbes, 1992; Vasileev, 1993; Munyua and Ngotho, 1998; Waruiru and Ngotho, 2001). Significant positive effects ranged from 40 to 50 more pounds gained (Duncan and Forbes, 1992; Vassilev, 1993), and there was an increase in average daily gain from 0.064 to 0.098 kg day^−1^ (Vassilev, 1993; Munyua and Ngotho, 1998) for ivermectin-only treatments, or up to 0.203 kg day^−1^ when clorsulon was added in the study using a sustained release bolus in calves (Waruiru and Ngotho, 2001). The 2 studies that followed cattle for periods less than 3 months post-treatment (Abdalla, 1989; Meeus et al. 1997) did not find a significant difference in growth rates. In the Meeus et al. study (1997), there was no untreated control group and the comparison was only among animals treated with various avermectins versus albendazole.

The remaining 4 studies included in the review were related to *Parafilaria bovicola*, a filarial parasite of cattle that causes subcutaneous lesions that resemble bruises and may progress to more extensive muscle involvement (Spickler, 2020). These lesions often result in significant profit losses for livestock owners due to the damage to hides and required muscle trimmings at slaughter. In all 4 studies, ivermectin showed a significant impact on lesion size and weight of trimmed tissue at the time of slaughter (typically above 90% reduction), when given as a single dose at least 70 days prior to slaughter as compared to untreated controls (Swan et al. 1983, 1991; Soll and Carmichael, 1984; Soll et al. 1991). In studies where some doses were given less than 70 days prior to slaughter (Soll and Carmichael, 1984), or when ivermectin was delivered orally (Swan et al. 1991), there was no or only partial improvement noted.

### Avermectin resistance

The 4 included studies were carried out in Kenya (*n* = 1), Cameroon (*n* = 1) and Nigeria (*n* = 2) (Table 2). Three of the studies were in beef cattle and 1 was in pigs. All of the studies were focused on the use of ivermectin against gastrointestinal helminths, and the animals were naturally infected prior to being enrolled in the studies. In 2 of the studies in cattle, ivermectin was delivered at the labelled dose of 200 mcg kg^−1^ SC (Idike et al. 2012; Mungube et al. 2015), with the third study comparing 200 mcg kg^−1^ SC to 1 mL/50 kg SC and 1 mL/50 kg SC with levamisole 7.5 mg kg^−1^ orally (Jean et al. 2016). In the single study in pigs, the dose was 300 mcg kg^−1^ SC (Idika et al. 2017). All studies employed the faecal egg count reduction test (FECRT) to determine resistance in the study population, which is recommended by the World Association for the Advancement of Veterinary Parasitology (WAAVP) in naturally infected animals (Geurden et al. 2022). The FERCT compares pre-treatment faecal egg counts with 14-day post-treatment faecal egg counts, and the WAAVP guidelines state that a greater than 90% reduction should be achieved to infer anthelmintic efficacy.

**Table 2.** Summary of studies (*N* = 4) assessing resistance to avermectins in cattle and swine in sub-Saharan Africa

Authors	Year	Title of study	Country	Type of avermectin, dose and delivery	Species	Parasite species identified	Measure of resistance	Results
Idika et al.	2012	Efficacy of levamisole and ivermectin in the control of bovine parasitic gastroenteritis in the sub-humid savanna zone of southeastern Nigeria	Nigeria	Ivermectin 200 mcg kg^−1^, SC	Cattle	*Haemonchus, Trichostrongylus, Cooperia* and *Bunostomum*	FECRT	100% reduction in faecal egg count for all cattle treated with ivermectin (no resistance).
Mungube et al.	2015	Prevalence of multiple resistant *Haemonchus* and *Ostertagia* species in goats and cattle in Machakos, eastern Kenya	Kenya	Ivermectin 200 mcg kg^−1^, SC	Cattle	*Haemonchus, Trichostrongylus, Ostertagia, Oesophagostomum* and *Cooperia*	FECRT	99% (95% CI: 91%–100%) reduction in faecal egg count when considering all helminth eggs; however, post-treatment faecal culture found 100% of remaining larvae were *Ostertagia* spp.
Jean et al.	2016	Efficacy testing of anthelmintics against field strains of *Trichostrongyles* in cattle farms of the periurban zone of Ngaoundere in Cameroon.	Cameroon	Ivermectin at 1 mL/50 kg SC; ivermectin at 200 mcg kg^−1^ SC and ivermectin 1 mL/50 kg SC with levamisole 7.5 mg kg^−1^ PO	Cattle	*Haemonchus, Trichostrongylus* and *Cooperia*	FECRT: arithmetic mean and geometric mean	Overall low levels of resistance were detected with the two groups that received ivermectin alone (primarily *Cooperia* spp.); however, the use of levamisole with ivermectin resulted in a 100% faecal egg count reduction.
Idika et al.	2017	Efficacy of ivermectin against gastrointestinal nematodes of pig in Nsukka area of Enugu State, Nigeria	Nigeria	Ivermectin 300 mcg kg^−1^ SC	Pigs	*Oesophagostomum dentatum, Ascaris suum* and *Trichuris suis*	FECRT	Ivermectin reduced *O. dentatum* eggs by 98.36% ± 0.43% and demonstrated a 100% reduction in *A. suum* and *T. suis.*

Ivermectin resistance was documented via the FERCT in one of the 4 included studies. The 2016 study in Cameroon found that ivermectin alone at either a 200 mcg kg^−1^ or 1 mL/50 kg dose produced FERCT results ranging from a 64% to 85% reduction with wide confidence intervals when using arithmetic means (Jean et al. 2016). Parasite species identified in this study were *Haemonchus, Trichostrongylus* and *Cooperia*. The study also found that when combined with levamisole, ivermectin given at 1 mL/50 kg was 100% effective. Although the 2015 study in Kenya found a 99% (95% CI: 91%–100%) reduction in faecal egg count when considering all helminth eggs, post-treatment faecal culture found 100% of remaining larvae were *Ostertagia* spp., which the authors interpreted as low or developing resistance in this particular species (Mungube et al. 2015). Based on the WAAVP guidelines, this would be best confirmed through a pre- and post-treatment coproculture, or potentially by using newer molecular-based tests. No evidence of resistance was found in the 2012 study in cattle or the 2017 study in pigs, both from Nigeria (Idika et al. 2012, 2017).

## Discussion

This restricted systematic review documents the paucity of research on the effects of avermectins on productivity outcomes in cattle and pigs in SSA. Among the 10 included studies, there is evidence that multiple doses of ivermectin do have a significant positive effect on weight gain in cattle when assessed over time periods greater than 3 months; however, only one study from Zimbabwe in 1992 linked this effect to a financial benefit for cattle-owners (Duncan and Forbes, 1992). In this case, cattle treated with ivermectin had a net advantage of 47 Zimbabwean dollars (ZWL) per head over the control group. For reference, the average income for that year was 4020 ZWL (World Bank, 2025). The 4 studies that investigated the use of ivermectin in cattle affected by the filarial parasite *Parafiliaria bovicola* showed marked efficacy against the parasite, resulting in reductions in lesion size. Two of the studies also documented a financial benefit due to the reduced trimming of subcutaneous and muscular tissue associated with lesion reduction. The 1982 study from Zimbabwe found an increase of 4.9 cents kg^−1^ (ZWL) paid at the time of slaughter for animals that received ivermectin at 70 days pre-slaughter, as compared to controls (Soll and Carmichael, 1984). The other study, performed in South Africa in 1991, found a difference in mean price realized per steer of 4.66 Rand between the treated and control groups, with a benefit-to-cost ratio of 4:1 (Soll et al. 1991).

Although the use of most avermectins is contraindicated in lactating dairy cows, studies in other regions of the world have documented the use of avermectins in cows during their reproductive dry period and have assessed interval from calving to conception and volume of milk production in the subsequent lactation cycle (Walsh et al. 1995; Gross et al. 1999). However, studies in SSA documenting other anticipated productivity outcomes, such as milk production or reproduction metrics, were not identified in this review. Additionally, no studies in swine that met inclusion criteria were identified.

Most of the 10 included studies were performed in the 1980s and 1990s, with the most recent study published in 2001, suggesting that perhaps other anthelmintics are now the focus of research in SSA or that related research is not published in journals included in the comprehensive databases selected for this review. Globally, there have been efforts to estimate and document the economic impact of parasites and their associated diseases in livestock (Rashid et al. 2019; Charlier et al. 2020; Strydom et al. 2023), but most are focused on intensive livestock systems and not small-holder herds, which may be another reason there are few studies in SSA. Extensive livestock production systems dominate in SSA, but measuring production-based outcomes within these systems can be challenging given the complex role that livestock play in the lives of 70% of the rural poor who depend on livestock or livestock-related activities for their livelihoods (Erdaw, 2023). However, if the use of ivermectin MDA in livestock for malaria vector control is proven effective, it offers an opportunity to consider how public health and veterinary sectors might collaborate for mutual benefit to the populations they serve. For example, the use of ivermectin MDA in cattle (for malaria vector control) would likely be repeated in multiple doses during the rainy season (i.e. the malaria season). Results of this review suggest that multiple doses will have a positive effect on cattle growth over time, but whether this would translate into financial benefit for owners is unknown. This is an example that demonstrates our need to better understand how the use of avermectins in smallholder livestock systems not only affects parasites, but how the effective treatment of parasites leads to quantitative changes in production metrics and subsequent economic impacts.

There is some evidence that resistance to ivermectin is developing in intestinal parasites of cattle in SSA (Kenya and Cameroon). However, the inclusion of only 4 studies in this review does not confirm widespread resistance, but rather a lack of investigation and documentation in cattle and swine parasites for the avermectins class, at least within the literature captured in the databases used in this study. Resistance to avermectins has been documented globally for decades across various species of livestock nematodes and ectoparasites. Mechanisms of resistance include alterations in ligand-gated ion channels and increased expression of ATP-binding cassette transporters, with multigenic mechanisms for resistance making it complex to understand and manage (Silvestre et al. 2011; Fissiha et al. 2021). Population-level resistance to anthelmintics typically occurs in under 10 years (Fissiha and Kinde, 2021), so in areas where avermectins have been used extensively and consistently, we would expect to find it.

Although there are no consistent data collected on avermectin access and use, Imbahale et al. (2019) mapped the areas in SSA where MDA for malaria vector control would potentially be best implemented, using overlapping maps of cattle density, zoophillic *Anopheles arabiensis* habitat and malaria prevalence. Areas identified include countries in the savanna region south of the Sahel in West Africa, and a scatter of areas within several countries in central and eastern SSA that are not dominated by rainforest or desert. An investigation or collaboration with national and local Veterinary Services within these areas would be critical to understanding the potential for existing avermectin resistance in areas where ivermectin MDA might be considered. This kind of collaboration would be valuable for the implementation of ivermectin MDA as well, as local veterinary personnel could assist in community engagement and lead drug delivery in livestock. With some innovative thinking, there could be options for cost savings and benefits across sectors and beyond malaria control, for example by combining MDA with livestock vaccination campaigns or working with NTD control programs.

There are 2 limitations to this study that should be noted. The first is that rapid systematic reviews are inherently limited by the extent of their search strategy. They are designed to quickly synthesize evidence on a particular topic and, in doing so, may leave out some relevant data. Therefore, inclusion of additional databases or grey literature to capture regionally relevant publications may provide further insight into the 2 questions posed in this study. Second, the search terms used did not include all possible avermectins. Although broad terms such as anthelmintic and avermectin were used, it is possible that some relevant studies were not returned in the results and, therefore, were not incorporated in this review. Despite these limitations, we believe this review provides a reasonable synthesis of peer-reviewed literature from which we can draw some conclusions.

## Conclusions

Despite the common assertion that control of endo- and ectoparasites in smallholder livestock systems would improve productivity outcomes, there actually exists little evidence to quantify this impact as it relates to the use of avermectins in cattle and swine in SSA. Ivermectin, the most commonly used of the avermectins, is readily available in many animal health pharmacies and feed stores throughout SSA. Although we may suspect that avermectin resistance would therefore be widespread, the literature does not currently support or refute this. As the public health community considers the use of ivermectin (or other avermectins) in livestock for malaria vector control, it becomes critical to better quantify potential benefits and risks within the animal health sector.
